# Are there any differences between adult-onset cerebellitis and childhood cerebellitis?

**DOI:** 10.1007/s10072-025-08127-5

**Published:** 2025-03-22

**Authors:** Rahşan Göçmen, Bahar Gülmez, Onur Ege Tarı, Aslı Tuncer

**Affiliations:** 1 Department of Radiology, Hacettepe University Faculty of Medicine, Ankara, Türkiye; 2 Department of Neurology, Hacettepe University Faculty of Medicine, Ankara, Türkiye

**Keywords:** Cerebellitis, Paraneoplastic cerebellar degeneration, Magnetic resonance imaging, Cerebellum, Inflammation

## Abstract

Acute cerebellitis (AC), a rare inflammatory disorder of the cerebellum, is primarily associated with infectious, post-infectious, and autoimmune mechanisms. It predominantly affects children and typically follows a benign and self-limited course. This study, the largest case series to date focusing exclusively on adult-onset AC, reveals distinct clinical, laboratory, and imaging characteristics compared to the pediatric AC literature. We included 16 patients (mean age of 39.5 years) who met the clinico-radiological features of AC. The causes of AC in this study were varied, encompassing paraneoplastic, post-infectious, infectious, anti-GAD-related, and hemophagocytic lymphohistiocytosis etiologies. The most common symptoms were altered consciousness, dysarthria, ataxia, vomiting, fever, headache and seizures. All cases showed bilateral cerebellar involvement on MRI, predominantly affecting the cerebellar cortex. Hemorrhage within cerebellar lesions was observed in three patients. Outcomes were often severe, with 56% of patients experiencing severe sequelae and 31% mortality. This study highlights the distinct characteristics and potential for severe outcomes in adult AC, emphasizing the need for a comprehensive diagnostic approach.

**Supplementary Information:**

The online version contains supplementary material available at 10.1007/s10072-025-08127-5.

## Introduction

Acute cerebellitis (AC), characterized by inflammation of the cerebellum, is a clinico-radiological syndrome. It manifests acute or subacute onset of cerebellar dysfunction along with radiological evidence of inflammatory swelling [1, 2]. It predominantly affects children and typically follows a benign, self-limited course. While the childhood version of this condition has been extensively studied, the adult-onset variant has received less attention. While the exact pathogenesis remains unclear, this entity is primarily associated with infectious, post-infectious and autoimmune mechanisms in children [3, 4]. While AC has been well-characterized in children, limited case reports and a lack of dedicated studies in adults have hindered a thorough understanding of its clinical features and outcomes in the adult population.

This retrospective study aims to explore the key differences between adult-onset cerebellitis and its childhood counterpart, including the underlying causes, clinical presentation, and prognosis.

## Materials and method

This study was performed in accordance with the Declaration of Helsinki and approved by the local ethics committee (protocol no: GO 22/618).

This single-center retrospective study included all patients over 18 years old admitted to our tertiary referral hospital between January 2010 and June 2024. While the terms acute cerebellar ataxia and acute cerebellitis have often been used interchangeably, a recent trend in the literature suggests differentiating them based on neuroimaging. Specifically, "acute cerebellitis" is increasingly being reserved for cases of acute cerebellar ataxia where MRI reveals inflammatory swelling in the cerebellum [1, 5]. Therefore, study patients had to meet the following inclusion criteria: presentation with symptoms compatible with acute/subacute cerebellar syndrome (e.g., ataxia, dysarthria, or nystagmus) or signs of inflammation on brain MRI. Patient demographics, clinical presentations, laboratory and imaging results, etiological diagnoses, treatment approaches, and outcomes were recorded (Supplementary Material 1).

The MRI studies were performed on 1.5 T or 3.0 T scanners. All but one patient underwent post-contrast imaging at diagnosis, and eleven had follow-up MRIs. A neuroradiologist, who was blinded to the clinical presentation and outcome, retrospectively reviewed the baseline and follow-up MRI examinations. The following criteria for the MRI study were evaluated: bilateral/unilateral, vermis, white matter, cortex, and dentate nucleus involvement; presence of narrowing of 4th ventricle, obstructive hydrocephalus, tonsillar herniation, and contrast medium enhancement.

Three patients, initially suspected of having AC based on clinical and radiological presentation, were excluded from the study due to subsequent diagnoses of mitochondrial disease, paraneoplastic cerebellar degeneration (without cerebellar inflammation or swelling), and Christianson syndrome-like features (Supplementary Material 1).

## Results

### Demographic and clinical characteristics

A total of 16 patients were included in the study. The mean age of the patients was 39.4 years (range: 19–83 years), with 62.5% of the patients being female. The most frequent symptoms experienced by the patients were altered consciousness (*n* = 12, 75%), dysarthria (*n* = 9, 56%), ataxia (*n* = 9, 56%), vomiting (*n* = 7, 44%), and fever (*n* = 7, 44%). Less common symptoms included seizures (*n* = 5, 31%) and headaches (*n* = 5, 31%). Most patients (75%, *n* = 12) experienced an acute onset of symptoms within 14 days, with an average duration of 3.8 days (standard deviation of 4.3 days, range 1–13 days). The remaining patients (*n* = 4) had symptom onset after 14 days. All cases of cerebellitis with infectious or post-infectious origins had an acute onset, while 75% of patients who developed subacute onset had paraneoplastic origin. The medical histories of the 16 patients were notable for the following: malignancy (*n* = 3), systemic infection (*n* = 1), immunosuppressant therapy (*n* = 4), autoimmune disorders (*n* = 2), pregnancy (*n* = 1), and no significant medical history (*n* = 6). Lymphopenia and leukocytosis were observed in 4 patients each. CSF pleocytosis (> 5 white cell count/μl) was recorded in 2/16 (12.5%). Lumbar puncture (LP) was performed in all but one patient. Despite the plan for a lumbar puncture (LP), one patient died due to rapidly progressing sepsis. Elevated protein levels were found in 5/15 (31.25%). OCB positivity was detected in 4/15 (%25). Elevated IgG index was present in 3/15 (18.75%). The etiologies of AC in the study population were distributed as follows: paraneoplastic (*n* = 4), postinfectious (*n* = 3), infectious (*n* = 3), unknown (*n* = 4), hemophagocytic lymphohistiocytosis syndrome (HLH) (*n* = 1), and autoimmune (anti-GAD antibody-related) (*n* = 1). The demographics, clinical features, laboratory results, treatments, etiologies, and outcomes are summarized in Table 1.

**Table 1:** Demographic, clinical and laboratory findings, treatment and outcome

Case	Age	Sex	Medical history	Symptoms	Disease onset (day)	CSF protein (mg/dL)	OCB	IgG index	Etiologic evaluation	Treatment	Follow up (months)	Etiology	Outcome
1	27	F	None	Altered consciousness, vomiting, dysarthria, ataxia, seizure	1	27	Type II	0.58	Microbiologic, autoimmune, and paraneoplastic tests: negative	IV Acyclovir, IVIg, IV steroid, oral steroid	10	Post-infectious	Stable/Mild or moderate neurological sequelae
2	19	M	Refractory epilepsy	Fever, altered consciousness, seizure	3	14	Type III	0.72	Influenza H1N1 positivity (in CSF)	IV Acyclovir, IVIg, IV steroid, oral steroid	6	Post-infectious	Progression / Severe neurological sequelae
3	26	M	None	Altered consciousness, vomiting, dysarthria	2	23	Negative	0.51	Microbiologic, paraneoplastic tests: negative	IV Acyclovir	12	Unknown	Stable / Severe neurological sequelae
4	45	F	Autoimmune polyglandular syndromes	Headache, fever, altered consciousness, dysarthria, ataxia	7	63	Type II	1.03	Autoimmune, paraneoplastic tests, solid malignancy: negative,	IV Acyclovir, IVIg, IV steroid	12	Autoimmune cerebellitis	Stable / Severe neurological sequelae
5	51	F	None	Headache, fever, vomiting, altered consciousness, dysarthria, ataxia, catatonia	1	23	-	0.52	Autoimmune, paraneoplastic tests: negative No solid malignancy Covid-19 Positive RT-PCR	IVIg, IV steroid	5	Post-infectious-SARS-CoV2	Stable / Severe neurological sequelae
6	31	F	28-week pregnancy	Dysarthria, dysmetry, ataxia, vomiting	7	45	-	0.73	Microbiologic tests: negative	IVIg	3	Unknown	Full recovery
7	44	F	Secondary CNS lymphoma	Altered consciousness, seizure	3	110	NK	NK	Cytomegalovirus serologic test positivity	IV Acyclovir	2	Infectious-CMV	Exitus
8	34	M	Marfan syndrome, aortic valve replacement (warfarin)	Dysarthria, dysmetry, ataxia, vomiting	12	33	-	1	Microbiologic, paraneoplastic tests, solid malignancy: negative	IV Acyclovir	10	Unknown	Exitus
9	23	F	Multiple liver abscesses	Altered consciousness, generalized tonic clonic seizure, vomiting	30	35	-	0.51	Microbiologic tests: negative	None	2	Unknown	Progression / Severe neurological sequelae
10	83	M	Metastatic colorectal cancer, Chemotherapy (cetuximab)	Altered consciousness, seizure	4	NK	NK	NK	NK	None	1	Paraneoplastic	Exitus
11	45	F	None	Headache, fever, vomiting, altered consciousness, dysarthria, ataxia	60	83	NK	NK	Paraneoplastic tests: anti-Yo/PCA1 antibody-positive Malignancy screening: breast cancer (invasive ductal carcinoma)	Plasma exchange	2	Paraneoplastic (AntiYo-Ab)	Progression / Severe neurological sequelae
12	43	F	None	Dysarthria, dysmetry, ataxia	20	67	Type II	1.31	Paraneoplastic tests: anti-Yo/PCA1 antibody-positive, Malignancy screening: breast cancer	IVIg	24	Paraneoplastic (AntiYo-Ab)	Stable / Severe neurological sequelae
13	39	F	None	Dysarthria, dysmetry, ataxia	90	23	-	0.48	Paraneoplastic tests: Anti-amphiphysin antibody-positive	IVIg	6	Paraneoplastic (Amphiphysin-Ab)	Stable / Severe neurological sequelae
14	29	M	Myasthenia gravis, received RTX 2 weeks ago	Headache, fever, vomiting, altered consciousness	13	11	-	0.56	Microbiologic tests: Enterovirus RNA PCR ( +)	IV Acyclovir, IVIg, plasma exchange	2	Infectious-Enterovirus	Progression / Severe neurological sequelae
15	25	M	Hematological malignancy, demyelinating peripheral neuropathy, IVIg, cyclosporine, etoposide, steroid for primary disease	Fever, muscle weakness, neuropathy	10	148	NK	NK	Microbiologic tests: negative, primary HLH gene test muation detected	None	4	Primary Haemophagocytic Syndrome	Exitus
16	66	F	Renal failure	Fever, altered consciousness	1	32	NK	NK	Microbiologic tests: negative	IV Acyclovir	2	Infectious	Exitus

*Ab* Antibody; *CMV* Cytomegalovirus; *CNS* Central nervous system; *CSF* Cerebrospinal fluid; *HLH* Hemophagocytic lymphohistiocytosis; *IgG* Immunoglobulin G; *IV* Intravenous; *IVIg* Intravenous immunoglobulin; *NK* Not known; *OCB* Oligoclonal bands; *PCA1* Purkinje cell antibody 1; *PCR* Polymerase chain reaction; *RTX* Rituximab; *SARS-CoV2* Severe acute respiratory syndrome coronavirus 2

### Magnetic resonance imaging findings

All cases demonstrated bilateral cerebellar involvement, with 14 exhibiting symmetrical and 2 exhibiting asymmetrical involvement. None had unilateral involvement. All but one case showed cerebellar vermis involvement. Cerebellar cortical involvement was observed in all patients. In 11 cases, this involvement was isolated to the cortex, while only 2 cases demonstrated predominant white matter involvement. Four cases had middle cerebellar peduncle involvement (one paraneoplastic, one infectious, one HLH, and one unknown etiology). Dentate nucleus involvement was present in three cases (two infectious, one HLH), while one case demonstrated the involvement of the mesencephalon (HLH-case) and another of the hippocampus (unknown etiology). Hemorrhage within the lesions was detected in three patients. Additionally, two patients with postinfectious etiology exhibited mild encephalitis/encephalopathy with a reversible splenial lesion (MERS). Pathological contrast enhancement was observed in eight cases, with five demonstrating parenchymal involvement and three exhibiting isolated leptomeningeal enhancement. Signs of increased intracranial pressure secondary to cerebellitis were observed in eight patients (50%), four of whom developed obstructive hydrocephalus. Cerebellar atrophy of varying degrees was present in 9 (3 subtle, 3 mild, 1 moderate, 2 severe) out of 10 patients who underwent follow-up imaging. In these cases, moreover, high intensities were noted in the affected cerebellar cortices on fluid-attenuated inversion recovery (FLAIR) images. The most rapid atrophy developed on the 18th day in a patient with Anti-GAD Ab-associated cerebellitis.

**Fig. 1:**
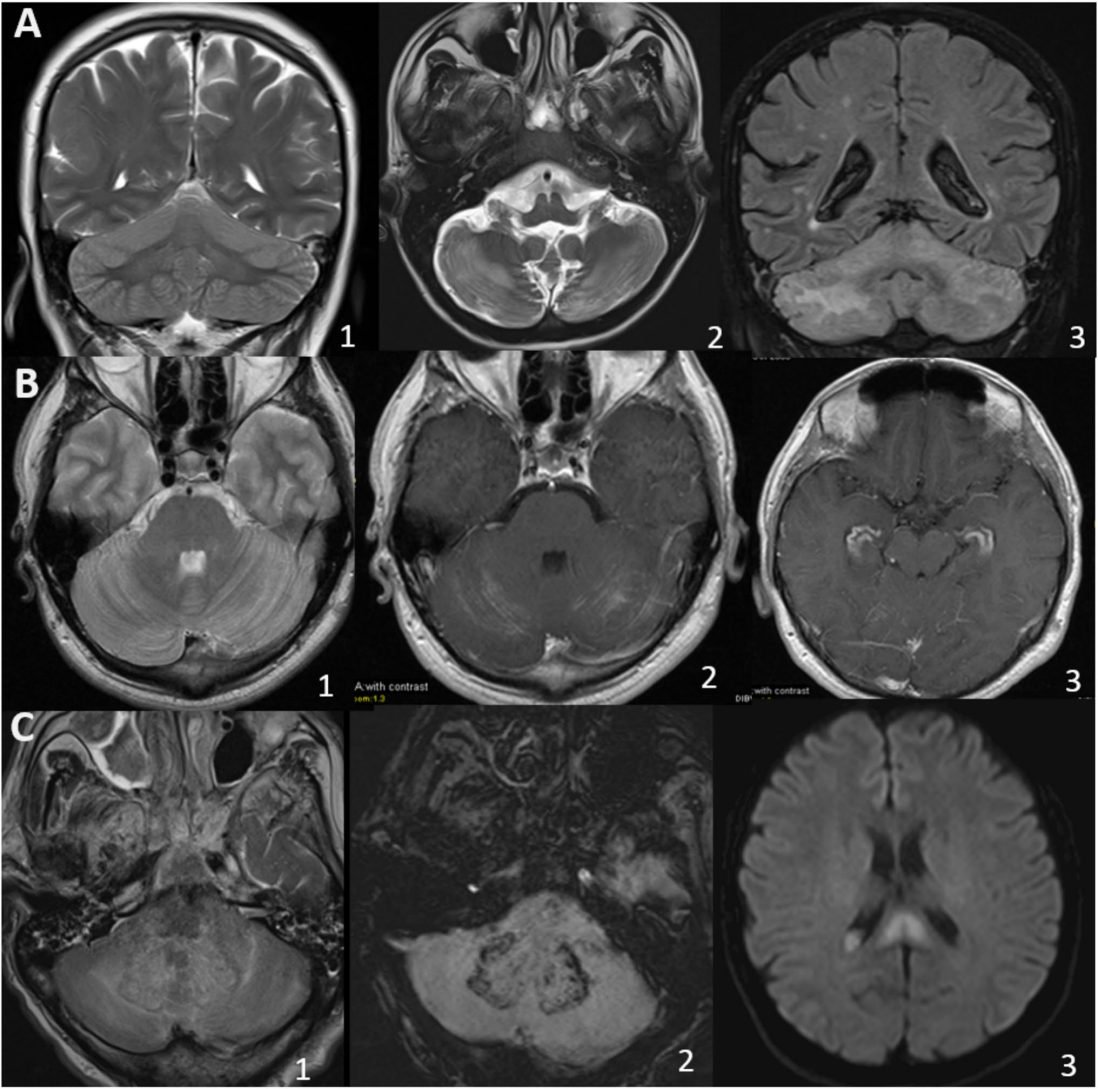
**A-C** Magnetic Resonance Imaging patterns of cerebellitis. Coronal and axial T2W images demonstrate involvement of the cortex only in Patient 1 with postinfectious cerebellitis (**A1**), predominantly cortex involvement in Patient 5 with postinfectious SARS-CoV2 cerebellitis (**A2**) and coronal FLAIR shows white matter predominantly involvement in Patient 15 with familial hemophagocytic lymphohistiocytosis (**A3**). Axial T2W image of Patient 3 reveals symmetrical cortical involvement (**B1**) and cortical contrast enhancement oriented parallel to the folia (**B2**). Notably, the same patient also exhibits bilateral hippocampal contrast enhancement (**B3**). Axial FLAIR (**C1**) and susceptibility-weighted image (**C2**) image of a patient with hemophagocytic lymphohistiocytosis demonstrate bilateral involvement of the dentate nuclei, and susceptibility-weighted image reveals hemorrhage within the dentate nuclei. Diffusion-weighted imaging of Patient 1 reveals a focal restricted diffusion in the splenium of the corpus callosum (**C3**). This finding is consistent with mild encephalopathy with reversible splenial lesion

Representative cases are demonstrated in Fig. 1, Supplementary Material 2 and radiological findings are summarized in Table 2.

**Table 2:** Radiological findings

Case	Location	Vermian involvement	Involvement of cortex/WM	Contrast enhancement	Findings of increased intracranial pressure				Imaging findings at follow-up		Length of follow-up	Other imaging findings
									Atrophy	Cortical FLAIR hyperintensity		
					Tonsillar herniation	Narrowing of 4th ventricle	Hydrocephalus	Enlarged optic nerve sheath				
1	BL-symmetrical	+	Cortex	LM	+	+	-	-	Subtle atrophy	+	42 months	MERS
2	BL-symmetrical	+	Cortex	-	-	-	-	-	Mild atrophy	+	0.5 months	
3	BL-symmetrical	+	Cortex	Parenchymal	+	+	-	-	N/A	+	6 months	Hemorrhagic, limbic encephalitis
4	BL-symmetrical	+	Cortex	LM	+	-	-	+	Severe atrophy	+	72 months	
5	BL	+	Cortex > WM	Parenchymal	+	+	+	+	Severe atrophy	+	0.26 months	MERS
6	BL-symmetrical	+	Cortex > WM	-	+	+	+	-	Subtle atrophy	+	84 months	
7	BL	+	Cortex	LM	-	-	-	-	Mild atrophy	+	10 months	
8	BL-symmetrical	+	Cortex	-	-	-	-	-	Mild atrophy	+	24 months	MCP
9	BL-symmetrical	+	Cortex	-	Mild	Mild	-	-	Moderate atrophy	+	6 months	
10	BL-symmetrical	+	Cortex	Parenchymal	-	-	-	-	N/A	N/A	N/A	
11	BL-symmetrical	+	WM > Cortex	Parenchymal	-	-	-	-	Subtle atrophy	+	10 months	MCP
12	BL-symmetrical	+	Cortex	-	-	-	-	-	N/A	N/A	N/A	
13	BL-symmetrical	+	Cortex	-	-	-	-	-	N/A	N/A	N/A	
14	BL-symmetrical	+	Cortex	-	+	+	+	+	-	-	18 months	Mesencephalon, MCP, VP shunt
15	BL-symmetrical	-	WM > Cortex	-	-	-	-	-	N/A	N/A		Hemorrhagic, cerebral-cerebellar atrophy
16	BL-symmetrical	+	WM = Cortex	Parenchymal	+	+	+	+	N/A	N/A	N/A	Hemorrhagic,dentate nucleus, MCP, VP shunt

*BL* Bilateral; *LM* Leptomeningeal; *MCP* Middle cerebellar peduncle; *MERS* Mild encephalitis/encephalopathy with reversible splenial lesion; *N/A* Not available; *WM* White matter; *VP* Ventriculoperitoneal

### Management and outcome

Nine patients were treated with intravenous immunoglobulin, intravenous methylprednisolone, oral prednisone, and plasma exchange immunotherapy, in order of frequency, either as monotherapy or combined. Four patients received only antiviral agent, while three patients did not receive any treatment. A ventricular drainage catheter was placed in two patients, while no patient received surgical decompression. Nine patients (56%) developed severe sequelae, with four of these patients becoming bedridden and requiring tracheotomy and gastrostomy. Five patients (31%) died, one patient (6%) had mild or moderate neurological sequelae, and only one patient (6.25%) made a full recovery. Sepsis due to hospital-acquired infections at the intensive care unit was the cause of death in three of the five patients; remaining two, the cause was unknown. The clinical follow-up period for the patients was a mean of 6.44 months (range: 1–24). The average survival time of the dead patients was 3.8 months (range: 1–10).

## Discussion

Our study, the largest case series to date focusing exclusively on adult-onset cerebellitis, provides valuable insights into the clinical, laboratory, and imaging characteristics of this rare condition. In contrast to the predominantly pediatric cerebellitis literature, our findings underscore the diverse etiology and potentially severe outcomes observed in adults. Acute cerebellitis in adults is very rare and only 35 cases have been reported between 1991 and 2017 [6].

AC usually occurs as a primary infectious prominently including varicella, influenza, rotavirus, human herpesvirus, Epstein-Barr virus, and Mycoplasma pneumoniae, postinfectious or much less post-vaccination reaction and mostly presents in early childhood [2–4, 7]. In line with prior adult AC cases, the heterogeneous etiologies identified in our study, including paraneoplastic, post-infectious, infectious, autoimmune causes, highlight the complexity of AC in adults [6]. This diversity necessitates a comprehensive diagnostic approach, incorporating detailed clinical history, laboratory investigations such as autoantibody screening, and imaging (including PET-CT). The cerebellum with its diverse cellular and antigenic composition is particularly susceptible to immune-mediated attacks due to the abundance of both extracellular and intracellular antigens [8]. Indeed, in our series, 10 out of 16 patients had immune-related cerebellitis, including paraneoplastic, post-infectious, anti-GAD antibody-associated, and HLH-associated cases. Notably, the identification of paraneoplastic cerebellitis in four patients emphasizes the importance of thorough oncologic evaluation in adults presenting with AC, particularly in the absence of an obvious infectious or autoimmune trigger. Three of them without a prior history of cancer were diagnosed with either breast cancer or lymphoma during their evaluation for cerebellitis, while one patient had a known history of colon cancer. In our study, anti-Yo and anti-amphiphysin antibodies, which were identified in patients with paraneoplastic AC, are among the major antibodies detected in paraneoplastic cerebellar degeneration (PCD) [9]. The design of this study included only patients with positive neuroimaging findings, as per the definition of "acute cerebellitis." Patients with PCD who were MRI-negative or presented with atrophy were excluded. Furthermore, in one of our cases with paraneoplastic AC syndrome, brain MRI findings were extremely subtle and questionable. A whole-body PET-CT scan, performed after detecting a positive Anti-Yo antibody, revealed increased cerebellar FDG uptake. In this patient, who was subsequently diagnosed with breast cancer, follow-up brain imaging revealed substantial cerebellar atrophy on MRI and decreased metabolic activity on PET-CT. This single case has been previously reported by Arslan et al. [10]. Paraneoplastic cerebellar degeneration/cerebellitis is a complex entity with various clinical scenarios possible: onconeural antibodies, MRI and malignancy positive [10, 11]; both onconeural antibodies and MRI positive, but no malignancy [12, 13]; both malignancy and MRI positive, onconeural antibodies negative [14] and lastly malignancy positive, MRI and antibodies negative but PET-CT positive [15]. Our findings further highlight that MRI and/or PET-CT abnormalities, such as increased signal intensity or hypermetabolism, may be detectable in the acute phase of paraneoplastic cerebellar degeneration, potentially before the onset of cerebellar atrophy.

Ataxia is the most common initial symptom in children with AC [2, 7]. Our study population similarly presented predominantly with dysarthria and ataxia. Notably, however, over half of our patients experienced altered consciousness, and seizures were observed in 31.25%, a frequency slightly higher than reported in the adult AC literature [6]. This could be attributed to the more widespread pathology or symptomatic seizures arising from underlying comorbidities and systemic infections. In our series, CSF abnormalities alone were not guide of a specific cause of AC. However, the presence of elevated CSF protein, oligoclonal bands, and an elevated IgG index suggests the need for a comprehensive evaluation for potential underlying causes, particularly in adult patients.

Consistent with studies in children with AC, our adult patients with infectious or post-infectious cerebellitis also presented with an acute onset, with the duration almost always being under 7 days [4]. However, our findings suggest that subacute onset may be associated with paraneoplastic cerebellitis. One young patient in our cohort was diagnosed with genetically-proven familial HLH after extensive investigations prompted by peripheral findings. To our knowledge, this is the first reported case of adult HLH presenting with acute cerebellitis (AC) in the literature.

Our study also highlights the utility of MRI in diagnosing and assessing the severity of AC, consistent with previous research [7, 16]. While unilateral cerebellar involvement is not uncommon in children with AC, all cases in our adult cohort exhibited bilateral involvement, with 87.5% demonstrating symmetrical involvement predominantly affecting the cerebellar cortex. Unilateral involvement and pseudotumoral hemicerebellitis, which are rare forms of acute cerebellitis in children, were not observed in our adult series [17]. However, the predominant involvement of the cerebellar cortex is consistent with previous reports in both adult and pediatric AC [7]. In line with previous studies on pediatric AC, vermian involvement was predominant in our adult cohort, observed in all but one case. [3, 7, 18]. Additionally, while white matter involvement was less common in our study, it is more frequently reported in children [3, 7, 18]. Interestingly, hemorrhage within cerebellar lesions was observed in three patients, a finding rarely reported in pediatric AC [19]. This suggests a potential difference in disease severity or pathophysiological mechanisms between adult and pediatric AC.

An intriguing concomitant finding in our study was the presence of MERS lesion in two patients with post-infectious AC (one of them had SARS-CoV2). This reversible splenial lesion, often associated with viral or post-viral infections, has been rarely reported to co-occur with cerebellitis, with only a few case reports documenting this association [20]. This co-occurrence suggests that reversible splenial lesion may serve as a potential marker of post-infectious etiology in patients presenting with AC. Nevertheless, further investigation is warranted to confirm the diagnostic utility of reversible splenial lesion in differentiating between various etiologies of AC.

Pediatric AC typically has a favorable clinical outcome despite radiological evidence of atrophy [7]. However, in both our series and previously reported cases, have shown that adult AC tends to have a less favorable prognosis [6]. One reason for the good outcome in children despite atrophy could be the plasticity and reserve capacity of the developing brain in children. While cerebellar atrophy with concomitant cortical T2-hyperintensity is often associated with conditions like infantile neuroaxonal dystrophy/ PLA2G6-associated neurodegeneration, Marinesco-Sjögren syndrome, congenital disorders of glycosylation type 1a, and certain mitochondrial disorders [21–23], our findings suggest that prior cerebellitis should also be considered in the differential diagnosis. All cases exhibiting atrophy in our cohort also displayed cortical hyperintensity. Yildirim et al. also reported this imaging feature in their pediatric AC case series [7].

There is no clear consensus or guideline to standardize the approach and management for AC. While steroids were prominent in childhood treatments [2], IVIg and combined steroid treatments stood out in our series. In contrast to the typically benign and self-limiting nature of AC in children, the condition proved less favorable in our adult cohort. Unfortunately, the majority of our patients experienced severe sequelae or died, resulting in a significantly higher rate of adverse outcomes compared to pediatric and adult case reviews, where sequelae rates are 25% and 50%, respectively [6, 7]. In healthy children, despite the absence of an underlying disease, the fulminant course of cerebellitis has been attributed to obstructive hydrocephalus and tonsillar herniation. However, the cause in the adult group remains unknown, and correlation studies have shown that this condition in adults is not related to radiological prognostic factors [24]. In our series, the high rate of adverse outcomes may be attributed to several factors. The underlying diseases and prior treatments of our patients could have negatively influenced their prognosis. Additionally, the extended follow-up period and the nature of our institution as a tertiary care hospital, which often manages patients with multiple comorbidities, might have contributed to the observed results. Calandrelli et al. found a significant correlation between radiological severity scores and clinical severity scales at the onset of AC symptoms [24]. However, they also noted that the radiological severity score did not predict the development of cerebellar atrophy or neurological sequelae in the later stages of the disease.

This study is subject to several limitations inherent to its retrospective, single-center design. The small sample size and lack of follow-up data for some patients. Despite these limitations, this study provides valuable insights into the clinical and radiological characteristics of cerebellitis in our adult patient population.

## Conclusion

Adult-onset cerebellitis presents a distinct clinical picture compared to its pediatric counterpart. Notably, post-infectious etiologies are less frequent in adults, with paraneoplastic and infectious origins being predominant. Furthermore, the initial presentation of cerebellitis may precede the detection of an underlying malignancy. While typically characterized by an acute onset in children, adult-onset cerebellitis may exhibit a more subacute course. Prognosis tends to be less favorable in adults. Consequently, the timely implementation of diagnostic evaluation and therapeutic intervention is of paramount importance to optimize patient outcomes.

## Supplementary Information

Below is the link to the electronic supplementary material.

Supplementary file1 (DOCX 57.5 KB)

Supplementary file2 (DOCX 1.18 MB)

## Data Availability

All data and material are available all of the authors to access. The datasets can be reached via hospital’s Picture, Archiving and Communications System (PACS) and central data system, named Nucleus.
