# Supplementary figures and images for: Are there any differences between adult-onset cerebellitis and childhood cerebellitis?
Source: Neurol Sci. 2025 Mar 22;46(7):3191–9. doi: 10.1007/s10072-025-08127-5 (PMC12152097; doi:10.1007/s10072-025-08127-5)

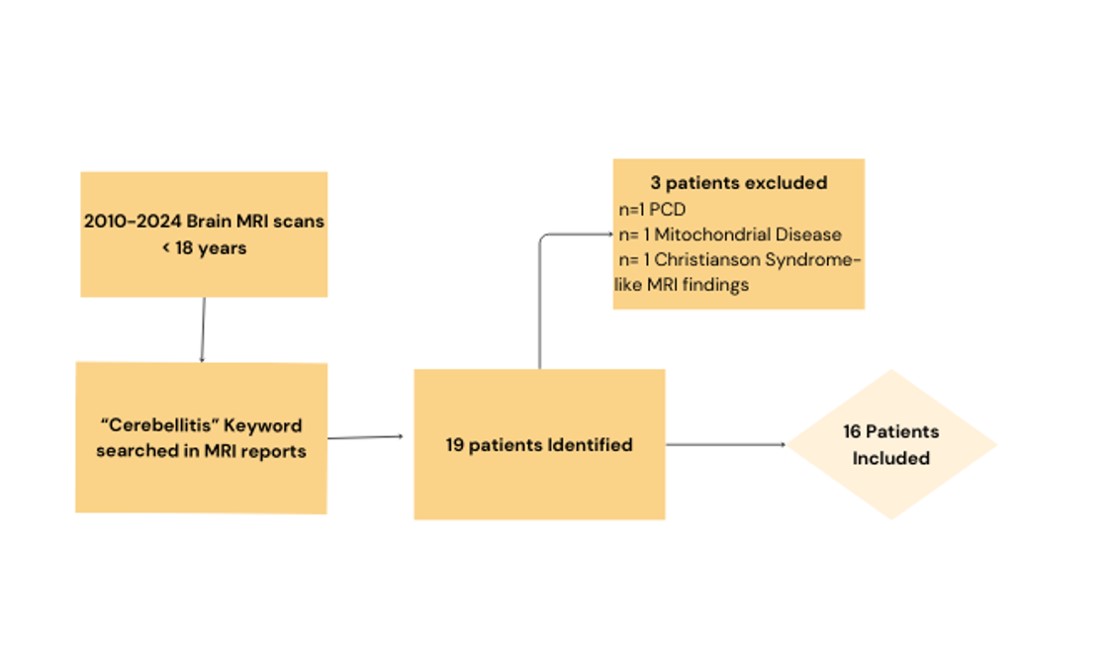


**Supplementary material 1:** Flow diagram for inclusion and exclusion of studies.

Supplement: Supplementary file 1 — Supplementary file1 (DOCX 57.5 KB) [file 10072_2025_8127_MOESM1_ESM.docx]
