# Supplementary material for: Are there any differences between adult-onset cerebellitis and childhood cerebellitis?
Source: Neurol Sci. 2025 Mar 22;46(7):3191–9. doi: 10.1007/s10072-025-08127-5 (PMC12152097; doi:10.1007/s10072-025-08127-5)
Supplement: Supplementary file 2 — Supplementary file2 (DOCX 1.18 MB) [file 10072_2025_8127_MOESM2_ESM.docx]

**
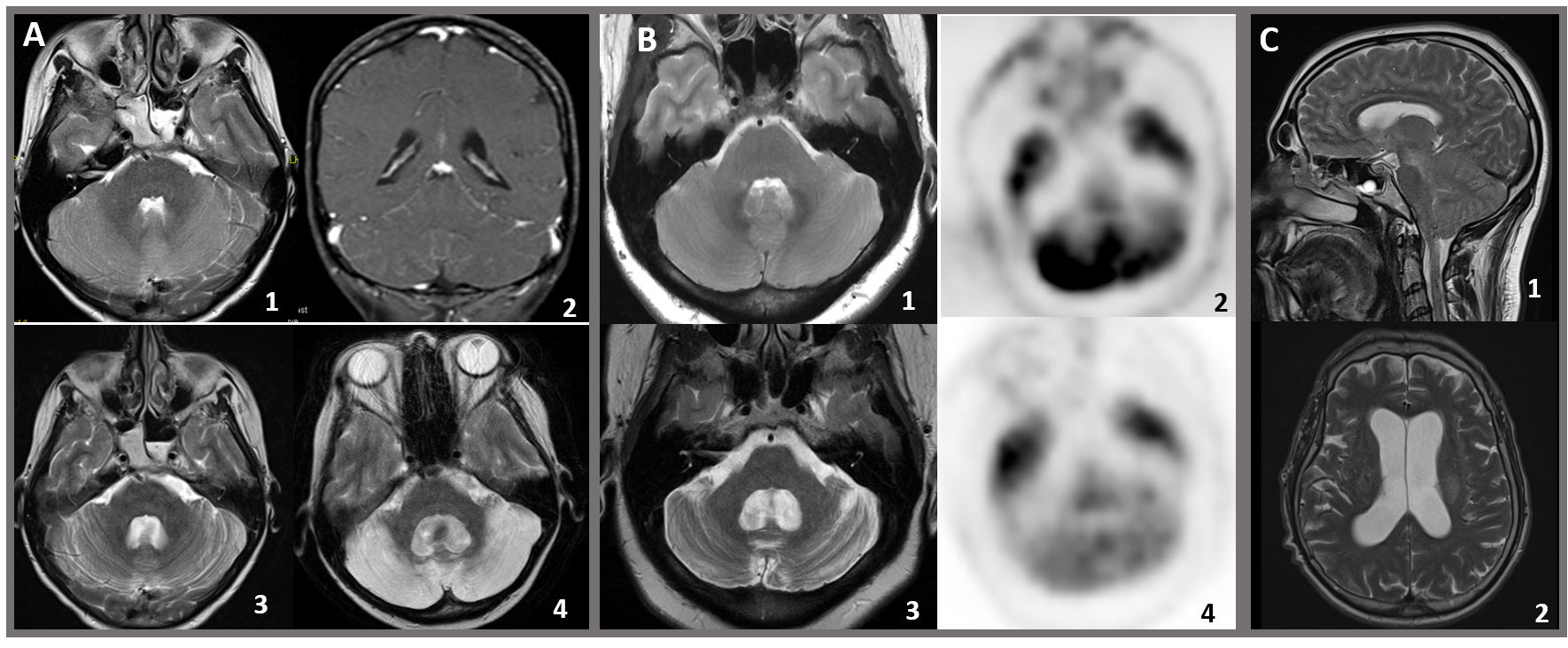
**

**Supplementary material 2 (A-C).** A. Serial MRI images of patient 4: Initial axial T2W image (A1) reveals bilateral, symmetrical cortical T2 hyperintensity with slightly narrowed 4th ventricle. Post-contrast coronal T1W image (A2) demonstrates increased leptomeningeal enhancement in the posterior fossa. Follow-up MRI at 17 days shows rapid cerebellar atrophy (A3)**.** 18 months later, cerebellar atrophy has progressed further with prominent cerebellar cortical T2 hyperintensity (A4). B. Axial T2W MRI image (B1) of patient 12 shows subtle questionable T2 hyperintensity. PET-CT (B2) performed 30 days later shows bilateral cerebellar hypermetabolism, more prominent on the right. MRI performed 1 year later reveals cerebellar atrophy (B3), and a corresponding PET-CT (B4) shows hypometabolism. C demonstrates complications of acute cerebellitis in two different patients: tonsillar herniation (C1) and hydrocephalus (C2).
